# Long-read individual-molecule sequencing reveals CRISPR-induced genetic heterogeneity in human ESCs

**DOI:** 10.1186/s13059-020-02143-8

**Published:** 2020-08-24

**Authors:** Chongwei Bi, Lin Wang, Baolei Yuan, Xuan Zhou, Yu Li, Sheng Wang, Yuhong Pang, Xin Gao, Yanyi Huang, Mo Li

**Affiliations:** 1 Laboratory of Stem Cell and Regeneration, Biological and Environmental Science and Engineering Division, King Abdullah University of Science and Technology (KAUST), Thuwal, 23955-6900 Kingdom of Saudi Arabia; 2 Present address: Key Laboratory of Zoonosis Research, Ministry of Education, Institute of Zoonosis, College of Veterinary Medicine, Jilin University, Changchun, China; 3 Computational Bioscience Research Center (CBRC), Computer, Electrical and Mathematical Science and Engineering (CEMSE) Division, King Abdullah University of Science and Technology (KAUST), Thuwal, 23955-6900 Saudi Arabia; 4 Beijing Advanced Innovation Center for Genomics (ICG), Biomedical Pioneering Innovation Center (BIOPIC), School of Life Sciences, College of Chemistry, College of Engineering, Peking-Tsinghua Center for Life Sciences, Peking University, Beijing, China; 5 Institute for Cell Analysis, Shenzhen Bay Laboratory, Shenzhen, China

**Keywords:** Human embryonic stem cell, CRISPR-Cas9, Genome editing, Nanopore sequencing, Long-read sequencing, Next-generation sequencing, Somatic mutation, Structural variant

## Abstract

Quantifying the genetic heterogeneity of a cell population is essential to understanding of biological systems. We develop a universal method to label individual DNA molecules for single-base-resolution haplotype-resolved quantitative characterization of diverse types of rare variants, with frequency as low as 4 × 10^−5^, using both short- or long-read sequencing platforms. It provides the first quantitative evidence of persistent nonrandom large structural variants and an increase in single-nucleotide variants at the on-target locus following repair of double-strand breaks induced by CRISPR-Cas9 in human embryonic stem cells.

## Background

Molecular consensus sequencing has been developed to enhance the accuracy of short-read next-generation sequencing (NGS) using unique molecular identifier (UMI) [1–3]. The use of UMI combined with bioinformatics enables the correction of random errors introduced by sequencing chemistry or detection. However, it remains challenging to analyze various types of genetic variants, because current methods are inadequate for detecting rare and/or complex variants (Additional file 1: Fig. S1). A case in point is the recent revelation that genome editing by CRISPR-Cas9 can lead to large deletions and complex rearrangements in various cell types, including mouse embryonic stem cells (mESCs) [4, 5]. It is unclear if this phenomenon also happens in human ESCs (hESCs) with identical characteristics, and more importantly, an unbiased and quantitative characterization of CRISPR-induced mutagenesis is still lacking due to limitation of current strategies.

Single molecule sequencing technologies can better resolve complex genetic variants by providing long reads [6], but they have a lower raw read accuracy [3]. To overcome these limitations, we have developed a strategy termed targeted Individual DNA Molecule sequencing (IDMseq). IDMseq guarantees that each original DNA molecule is uniquely represented by one UMI group (a set of reads sharing the same UMI) after sequencing, thus preventing false UMI groups and allowing quantification of allele frequency in the original population (Additional file 1: Fig. S1 & S2a). It is designed to be adaptable to various sequencing platforms and combines error correction by molecular consensus with long-read sequencing, thus enabling sensitive detection of all classes of genetic variants, including single nucleotide variants (SNVs), indels, large deletions, and complex rearrangements.

## Results

### IDMseq can detect rare subclonal variants

To verify that IDMseq can detect subclonal variants below the sensitivity limit of NGS (~ 1% [7, 8]), we constructed synthetic cell populations harboring a mutation at various pre-determined allele frequencies. We knocked in a homozygous SNV in the *EPOR* gene using CRISPR-Cas9 in the H1 hESCs (Additional file 1: Fig. S3a-c). A rare subclonal mutation in a population of cells is simulated by admixing the genome of knock-in and wild-type cells at different ratios.

First, we tested if IDMseq could overcome the high base-calling error of Nanopore sequencing in rare mutation detection. A 168-bp stretch of DNA encompassing the knock-in SNV was labeled with UMIs and amplified from a population with the ratio of 1:100 between knock-in and wild-type alleles. We developed a bioinformatics toolkit called Variant Analysis with UMI for Long-read Technology (VAULT) to analyze the sequencing data (Additional file 1: Fig. S2b; see the “Methods” section). The results showed that 36.5% of reads contained high-confidence UMI sequences (Table 1). Based on a pre-set threshold of a minimum of 5 reads per UMI group, those reads are binned into 284 UMI groups. It is worth noting that every UMI group represents an original allele in the genome of the initial population. VAULT analysis showed that 2 UMI groups contained the knock-in SNV (Additional file 1: Fig. S4a). Furthermore, no spurious mutation was detected. Importantly, when the trimmed reads were pooled for variant analysis without considering UMIs, no variant could be detected by the same algorithms, proving the superior sensitivity afforded by IDMseq. These results suggest that IDMseq on the single-molecule Nanopore sequencing platform is able to accurately call rare variants without false positives.

**Table 1:** Summary of individual sequencing runs

Gene	Mutant allele frequency (%) / Type	Amplicon size	Sequencing platform	Read count	Reads with UMI	UMI groups for variant calling (≥ 5 reads)	Median read number per UMI group	UMI groups with introduced mutation	Somatic SNV count	Somatic SNV load per megabase	SV groups
EPOR	1:100 (1%)	168 bp	Nanopore	17,634	6444	284	7	2 (0.7%)	0	N/A	N/A
EPOR	1:1000 (0.1%)	6789 bp	PacBio	227,206	136,399	3184	6	4 (0.126%)	192	9.0	3
EPOR	1:10,000 (0.01%)	168 bp	Nanopore	1,093,683	494,009	15,598	8	1 (0.006%)	10	7.1	N/A
EPOR	1:10,000 (0.01%)	168 bp	Illumina	7,488,257	7,236,007	132,341	7	5 (0.004%)	85	7.1	N/A
PANX1 (Pan1)	WT	7077 bp	Nanopore	576,583	165,628	2810	6	N/A	73	3.8	0
PANX1 (Pan3)	WT	6595 bp	Nanopore	389,726	133,215	3867	7	N/A	103	4.1	0
PANX1 (Pan1)	Cas9 editing	7077 bp	Nanopore	2,761,805	613,147	3479	7	N/A	275	11.3	189 (5.4%)
PANX1 (Pan3)	Cas9 editing	6595 bp	Nanopore	3,078,165	1,042,582	7281	10	N/A	624	13.1	204 (2.8%)

**Fig. 1:**
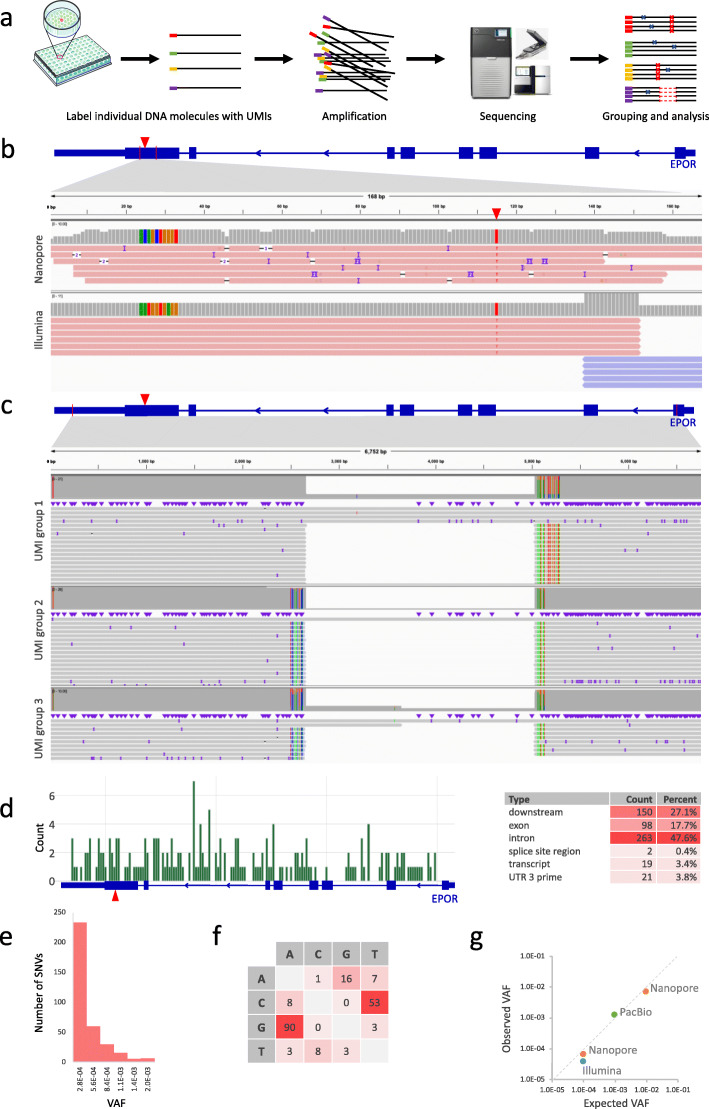
IDMseq for detection of subclonal variants. **a** Schematic representation of IDMseq. Individual DNA molecules are labeled with unique UMIs and amplified for sequencing on appropriate platforms (e.g., Illumina, PacBio, and Nanopore). During data analysis, reads are binned by UMIs to correct errors introduced during amplification and sequencing. Both SNV and SV calling are included in the analysis pipeline. **b** Examples of Integrative Genomics Viewer (IGV) tracks of UMI groups in which the spike-in SNV in the 1:10,000 population was identified by IDMseq and VAULT. The knock-in SNV is indicated by the red triangle in the diagram of the *EPOR* gene on top, and also shown as red “T” base in the alignment map. The gray bars show read coverage. The ten colored bars on the left side of the coverage plot represent the UMI sequence for the UMI group. Individual Nanopore (top) and Illumina (bottom) reads within the group are shown under the coverage plot. **c** Large SVs detected by IDMseq in the 1:1000 population on the PacBio platform. Three UMI groups are shown with the same 2375-bp deletion. Group 1 represents one haplotype, and groups 2 and 3 represent a different haplotype. Colored lines represent the SNPs detected in each group. Thick blue boxes: exons; thin blue boxes: UTRs. Thin vertical red lines in the gene diagram represent PCR primer location. **d** Distribution of SNVs detected by PacBio sequencing in conjunction with IDMseq and VAULT. One of the SNVs was also found in the Nanopore dataset. The spike-in SNV (1:1000) is indicated by the red triangle. The table on the right summarizes the frequency of SNV-associated records in different annotation categories. The numbers in the table represent annotation records from all transcript isoforms, so the same SNV may be recorded more than once. **e** Frequency distribution of the variant allele fraction of SNVs detected by IDMseq in PacBio sequencing of the EPOR locus. **f** The spectrum of base changes among somatic SNVs. The majority of base changes are G to A and C to T. **g** Comparison between observed VAF and expected VAF in different experiments and sequencing platforms

Detection of rare variants in clinical settings often demands sensitivities well below that of prevailing NGS platforms (ca. 10^−2^). For instance, early cancer detection using circulating tumor DNA is estimated to require a sensitivity of at least 1 in 10,000 [9]. To simulate this scenario, we next sequenced the same 168-bp region in a population with the ratio of 1:10,000 between knock-in and wild-type alleles (Fig. 1a). It is worth noting that the UMI-labeling reaction contained only around 5 copies of the knock-in allele. A 48-h sequencing run on the MinION acquired 1.1 million reads (Additional file 1: Fig. S4b). VAULT showed that 45.2% of reads contained high-confidence UMI sequences (Table 1). These reads were binned into 15,598 UMI groups (Additional file 1: Fig. S4c) of which one (0.6 × 10^−4^) contained the knock-in SNV (Fig. 1b). Ten other SNVs were also identified in ten UMI groups. We considered if these were PCR artifacts, as the main source of errors in UMI consensus sequencing originates from polymerase replication error in the barcoding step [10]. The Platinum SuperFi DNA polymerase we used has the highest reported fidelity (> 300X that of *Taq* polymerase). It not only significantly reduces errors in the barcoding and amplification steps, but also captures twice more UMIs in the library than *Taq* [10]. Theoretically, Platinum SuperFi polymerase introduces ~ 6 errors in 10^6^ unique 168-bp molecules in the UMI-labeling step. Accordingly, this type of inescapable error is expected to be around 0.09 in 15,598 UMI groups, and thus cannot account for the observed SNV events. This lets us to conclude that the ten SNVs are rare somatic mutations that reflect the genetic heterogeneity of hESCs as described previously [11]. These data provided an estimate of 7.1 somatic SNVs per megabase (Mb), which is consistent with the reported frequency of somatic mutation in coding sequence in normal healthy tissues [12].

The length of the 168-bp amplicon also allowed benchmarking against the industry standard Illumina sequencing, which features shorter reads but higher raw-read accuracy. We then sequenced the same 1:10,000 mixed population on an Illumina MiniSeq sequencer and obtained 7.5 million paired-end reads (Fig. 1a and Additional file 1: Fig. S4b). The results showed that 96.6% of reads contained high-confidence UMI sequences that were binned into 132,341 UMI groups (Additional file 1: Fig. S4c), in which 5 (4 × 10^−5^) contained the knock-in SNV (Table 1, Fig. 1b). The Illumina sequencing detected 85 somatic SNVs, of which seven overlapped with the ten (70%) detected by IDMseq using Nanopore sequencing. These overlapping SNVs were identified in multiple UMI groups (between 3 and 11) in Illumina sequencing, while the three non-overlapping SNVs were each discovered in one UMI group in Nanopore sequencing. Since IDMseq sequences individual original molecules, it necessitates that the Illumina and Nanopore experiments sequenced two distinct subsets of the original pool of molecules. It is possible that these three SNVs had lower actual allele frequencies and happened to be present in the subset of original molecules that went into the Nanopore library but not the Illumina one. As with any high-throughput sequencing method, the accuracy of allele frequency estimate improves with sequencing depth. Because of the high cost of Nanopore sequencing, it was performed at a depth that was enough to analyze the knock-in SNV (approximately 1/8 of the depth of the Illumina sequencing). However, this sequencing depth might not be enough for the analysis of ultra-rare somatic mutations, so these Nanopore somatic mutation data should be interpreted with caution. Nevertheless, the overall calculated somatic SNV load in the Illumina sequencing was 7.1 per Mb, which closely matched the Nanopore data (Table 1).

We next applied IDMseq to a larger region (6789 bp) encompassing the knock-in SNV in a population with 0.1% mutant cells on a PacBio platform (Fig. 1a and Additional file 1: Fig. S4b). VAULT showed that 60.0% of high-fidelity long reads contain high-confidence UMIs, binned into 3184 groups (Additional file 1: Fig. S4c). Four UMI groups (1.26 × 10^−3^) contained only the knock-in SNV. Another 186 groups contained 273 SNVs (174 groups with 1 SNV, 9 groups with 2 SNVs, and 3 groups with 27 SNVs, Table 1). Again, polymerase error during barcoding (~ 0.82 error in 3184 UMI groups) cannot account for the observed SNVs, suggesting that most SNVs are true variants. Interestingly, structural variant (SV) analysis showed that the three groups with 27 SNVs shared the same 2375-bp deletion. Haplotyping using the SNVs revealed that the three groups came from two haplotypes (Fig. 1c). This large deletion is far away from the Cas9 target site and thus less likely the result of genome editing. After excluding the SNVs in the large-deletion alleles, the remaining 192 SNVs distributed evenly in the region (Fig. 1d). Functional annotation of the SNVs showed that 17 of 192 caused an amino acid change. The spectrum of base changes and distribution of variant allele frequency (VAF) are consistent with published work [12] (Fig. 1e, f). These data provide an estimate of about 9.0 somatic SNVs per Mb.

Taken together, these data showed that IDMseq provides reliable detection of rare variants (at least down to 10^−4^) and accurate estimate of variant frequency (Fig. 1g). It is useful for characterizing the spectrum of somatic mutations in human pluripotent stem cells (hPSCs). Furthermore, it revealed a previously unappreciated phenomenon of spontaneous large deletion in hPSCs. Due to its large size and low frequency (VAF ≈ 0.1%), this SV would have been missed by short-read sequencing or ensemble long-read sequencing. Yet, it is conceivable that such an SV could confer growth advantage to the cells carrying it, and therefore has implications for the safety of hPSC in clinical settings. These findings clearly demonstrate the power of the combination of long-read sequencing and IDMseq in resolving complex genetic heterogeneity.

### IDMseq enables quantitative analysis of DNA repair outcomes in Cas9-edited hESCs

Despite its widespread adoption as an efficient and versatile genome-editing tool, the impact of the CRISPR-Cas9 system on human genome integrity remains poorly understood [4, 13, 14]. Previous work indicated that the most prevalent DNA repair outcomes after Cas9 cutting are small indels (typically < 20 bp) [15, 16]. Unexpectedly, recent studies revealed large and complex SVs over several kilobases represent a significant portion of the on-target mutagenesis effect of Cas9 [4, 5]. This phenomenon has been reported in a few cell types, including mESCs, but it remains to be characterized in hESCs. Importantly, to date, the analysis of large-deletion alleles came either from ensemble amplicon sequencing [4, 5] or whole-genome sequencing [5]. The former is prone to amplification bias, and the latter cannot adequately detect large and complex variants due to the limited read length. Thus, we applied IDMseq to wild-type (WT) hESCs and hESCs following CRISPR-Cas9 editing, to offer an unbiased quantification of the frequency and molecular feature of the DNA repair outcomes of double-strand breaks induced by Cas9.

**Fig. 2:**
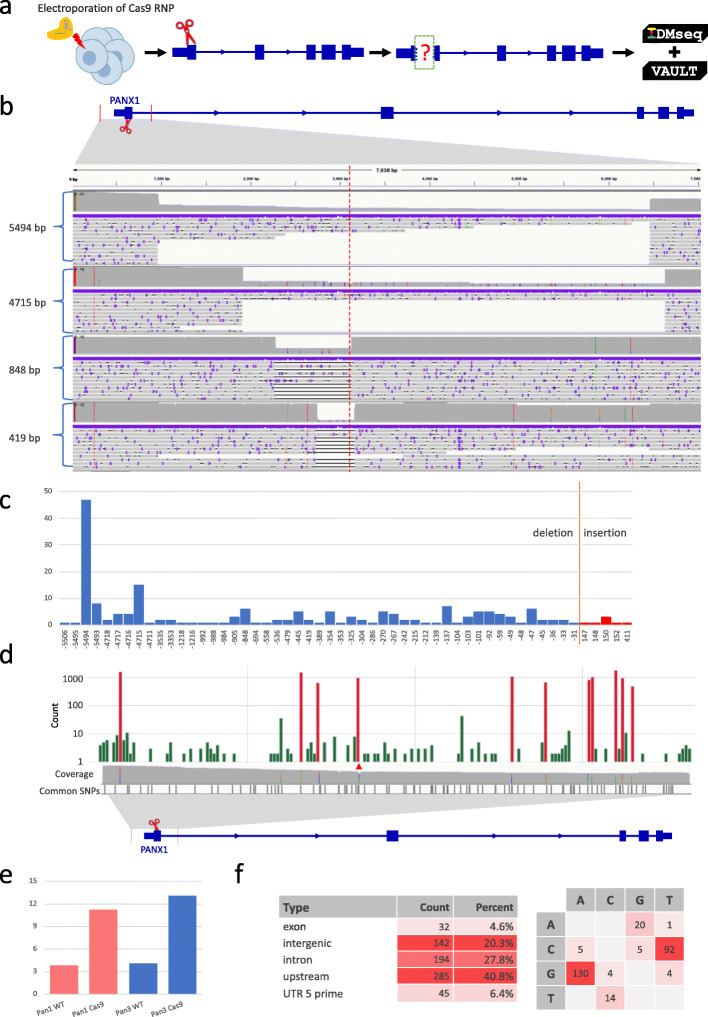
Quantitative analysis of DNA repair outcome of Cas9-induced DNA double-strand break in hESCs. **a** Schematic representation of the experimental design. Cas9 RNPs designed to cleave the first exon of *PANX1* were electroporated to H1 hESCs. IDMseq was used to analyze the locus in edited hESCs 48 h later. **b** Large SVs detected by IDMseq and VAULT in edited hESCs. Five SV groups were shown with deletion length ranging from 419 to 5494 bp. The red dotted line represents the Cas9 cutting site. The coverage of Nanopore reads is shown on top of each track in gray. The colored lines on the left side of the coverage plot represent the UMI for the group. Individual Nanopore reads within the group are shown under the coverage plot. **c** The frequency of deletions or insertions of different size detected in Pan1-edited hESCs. Certain deletions and insertions occur at disproportionally high frequencies. For example, a 5494-bp deletion was found in 56 UMI groups, which indicates a possible hotspot of Cas9-induced large deletion. **d** Distribution of SNVs detected by IDMseq and VAULT in Pan1-edited hESCs. Somatic SNVs are shown in green, while the cell-line specific SNVs are shown in red (using 40 bp of bin size in the figure). Somatic SNVs cannot be detected if variant calling is done en masse without UMI analysis (see the coverage track). Cell-line specific SNVs are detected in ensemble analysis (see colored lines in the coverage track) and most of them have been reported as common SNPs in dbSNP-141 database (common SNP track). The Cas9 cut site is indicated by the red triangle. **e** The number of presumed somatic SNVs per Mb (*y*-axis) in *PANX1* WT and Cas9-edited cells. **f** Analysis of somatic mutations detected in Pan1-edited hESCs based on functional annotation and base change. The majority of base changes are G to A and C to T

We targeted exon 1 (Pan1) and exon 3 (Pan3) of the Pannexin 1 (*PANX1*) gene with two efficient gRNAs (Fig. 2a). Forty-eight hours after electroporation of Cas9 complexed with the Pan1 or Pan3 gRNA, H1 hESCs were harvested for IDMseq. WT H1 hESCs cultured in parallel were used in the control sequencing. The surveyed region is 7077 bp for Pan1 and 6595 bp for Pan3. A 48- h Nanopore sequencing run of Cas9-edited cells yielded 2.8 million and 3.1 million reads for Pan1 and Pan3, which were binned into 3479 and 7281 UMI groups, respectively (Table 1, Additional file 1: Fig. S5a and b). For the sequencing run of WT cells, we obtained 2810 and 3867 UMI groups for Pan1 and Pan3, respectively (Table 1).

We first surveyed SVs (> 30 bp) in UMI groups. No SVs were detected in the sequencing of WT cells. For Cas9-edited cells, after SV calling and filtering out lowly supported SVs (see the “Methods” section), 189 (5.4%) of the 3479 UMI groups contained 191 SVs in Pan1-edited cells, including 184 deletions and 7 insertions. The size of SVs ranged from 31 to 5506 bp (Fig. 2b and c). Intriguingly, some large deletions were independently captured multiple times. For example, 47 (24.9%) UMI groups have the same 5494-bp deletion and 15 (7.9%) UMI groups have the same 4715-bp deletion. For the insertion variants, 3 of the 7 UMI groups shared the same SV (Fig. 2c).

When a different gRNA (Pan3) was used, 204 (2.8%) of 7281 UMI groups contained 211 SVs (164 deletions, 39 insertions, and 8 inversions), with size ranging from 31 to 4238 bp (Additional file 1: Fig. S6a). Importantly, reoccurring SVs were also detected with Pan3. For example, twenty-five (12.3%) UMI groups shared the same 4238-bp deletion, and 4 (2.0%) groups shared a 2750-bp insertion (Additional file 1: Fig. S6a). These data provided the first quantitative evidence that the repair outcomes of Cas9 cutting may not be random and there are likely hotspots for Cas9-induced large deletions or insertions.

We next analyzed SNVs in these data sets. WT and Cas9 editing with the Pan1 and Pan3 gRNAs resulted in similar SNV patterns (Fig. 2d, Additional file 1: Fig. S5g, and S7a-b). Specifically, the results of Pan1-edited cells showed that 2709 (77.9%) of 3479 UMI groups contained 11,861 SNPs, while for Pan3-edited cells 6986 (95.9%) of 7281 UMI groups contained 23,329 SNVs. In all cases, the SNVs fell into two frequency ranges. Most SNVs in the high-frequency category (red in Fig. 2d, Additional file 1: Fig. S5g, and S7a-b) have been reported in the common SNP database. The low-frequency SNVs (green in Fig. 2d, Additional file 1: Fig. S5g, and S7a-b) distributed evenly in the locus and did not overlap with known SNPs, likely representing somatic mutations. It is worth noting that the number of presumed somatic SNVs increased about 300% after Cas9 editing in both Pan1 and Pan3 regions, and the frequency of somatic SNVs increased from 3.8 to 11.3 and 4.1 to 13.1 per Mb for Pan1 and Pan3, respectively (Fig. 2e). In Cas9-edited cells, there was no obvious enrichment of SNVs immediately adjacent to the cutting sites, which is consistent with previous reports [17]. The spectrum (Fig. 2f, Additional file 1: Fig. S6d and S7c-d) and VAF (Additional file 1: Fig. S6b-c and S7c-d) of single nucleotide substitutions were consistent with published data [18].

For reasons not immediately clear, Nanopore sequencing of WT cells generated less reads than that of Cas9-edited cells despite using twice as many flow cells. To rule out the possibility that the observed differences in SNVs and SVs were due to sequence depth biases, we matched the sequencing depth of WT and Cas9-edited samples by randomly sampling reads in Cas9-edited samples (Additional file 1: Table S1, Fig. S8). The same WT libraries were sequenced in two batches (Batch 1 and Batch 2, Additional file 1: Table S1). For Cas9-edited cells, the numbers of subsampled reads were set to match the corresponding raw read numbers of WT Batch 1 (the 5th column of Additional file 1: Table S1), the numbers of reads with UMI of WT Batch 1 (the 6th column of Additional file 1: Table S1), or the numbers of reads with UMI of WT Batch 1 + Batch 2. All of the random subsamplings were performed more than 100 times. The results of 623 subsampling experiments showed that our original observation of a significant increase in the number of somatic SNVs and SVs around the cleavage site after Cas9 editing remained robust (Additional file 1: Table S1). The subsampling experiments showed small variations in the estimated somatic SNV load per Mb and SV frequency, which might be due to the stochasticity of UMI groups with low coverage meeting the stringent quality filter (see the “Methods” section). The accuracy of allele frequency estimate could be further enhanced by sequencing deeper, as with any high-throughput sequencing method, or by improving base-calling accuracy of Nanopore sequencing, which would in effect increase the number of reads with UMI. Nonetheless, these data from real-world and in silico experiments ruled out any artifact due to sequencing depth biases and validated the increase of somatic SNVs and SVs near the Cas9 cut site following Cas9 editing.

Besides SNVs and SVs, VAULT also reported many small indels around the Cas9 cleavage site. We compared the indels with the Sanger sequencing data of single-cell derived hESC clones. The results showed that IDMseq correctly identified a subset of the deletion alleles (Additional file 1: Fig. S5c-f).

## Discussion

In this study, we developed IDMseq and VAULT to enable quantitation and haplotyping of both small and large genetic variants at the subclonal level. They are easy to implement and compatible with all current sequencing platforms, including the portable Oxford Nanopore MinION sequencer. As compared to another long-read targeted sequencing technique named nCATS [19], which is able to survey multiple loci simultaneously, IDMseq shows several additional advantages including high capture efficiency, low input requirement, and high accuracy (Additional file 1: Table S2). On the other hand, nCATS, being PCR-free method, can detect DNA modifications, which are unfortunately lost in the targeted amplification of IDMseq. In this study, we showed evidence of increased somatic SNVs and reoccurring large SVs in Cas9-edited hESCs using two independent gRNAs in the same locus. It will be important to apply the methods described here to additional loci in future studies to confirm these observations and to obtain a more compete landscape of such intrinsic gene-editing features. IDMseq in its current form only sequences one strand of the DNA duplex, and its performance may be further improved by sequencing both strands of the duplex.

## Conclusions

IDMseq provides an unbiased single-base-resolution characterization of on-target mutagenesis induced by CRISPR-Cas9, which could facilitate the experimental design and safe use of the CRISPR technology in the clinic. Our results show that IDMseq is accurate in profiling rare somatic mutations, which can aid the study of genetic heterogeneity in pluripotent and somatic stem cells and can be further expanded to many other applications for quantitative assessments of genomic variations.

## Methods

### Generation of the knock-in hESC line

The H1 hESC line was purchased from WiCell and cultured in Essential 8™ medium (ThermoFisher) on hLaminin521 (ThermoFisher) coated plate in a humidified incubator set at 37 °C and 5% CO2. Electroporation of Cas9 RNP was done using a Neon Transfection System (ThermoFisher) using the following setting: 1600 v/10 ms/3 pulses for 200,000 cells in Buffer R (Neon Transfection kit) premixed with 50 pmol Cas9 protein (CAT#M0646T, New England Biolabs), 50 pmol single guide RNA (sgRNA), and 30 pmol single-stranded oligodeoxynucleotides (ssODN, purchased from Integrated DNA Technologies, Inc.) template. After 48 h, single cells were collected and seeded at 1000 single cells per well (6-well format). Seven days later, single colonies were picked for passaging and genotyping. The *EPOR* sgRNA sequence including protospacer adjacent motif (PAM) is 5′GCTCCCAGCTCTTGCGTCCA-TGG(PAM)3′, which was synthesized in vitro by MEGAshortscript™ T7 Transcription Kit (ThermoFisher).

### CRISPR-Cas9 editing of hESCs

CRISPR-Cas9 editing of the PANX1 locus in H1 hESCs were performed in the same way as the generation of knock-in hESCs except for the omission of the ssODN template. After 48 h, cells were collected for the genome extraction and library preparation. The Pan1 sgRNA sequence is 5′ATCCGAGAACACGTACTCCG-TGG(PAM)3′, and Pan3 sgRNA is 5′GCTGCGAAACGCCAGAACAG-CGG(PAM)3′.

### UMI primer design

The UMI primer contains a 3′ gene-specific sequence, a UMI sequence, and a 5′ universal primer sequence. The 3′ gene-specific sequence was designed with the same principle as PCR primers. We chose the sequence with an annealing temperature higher than 65 °C to improve specificity to the target gene. The internal UMI sequence consists of multiple random bases (denoted by Ns). The number of random bases is determined by the number of targeted molecules. We chose a short UMI sequence (10–12 nt) to reduce the sequencing errors within the UMI. We adopted a unique sequence structure in the UMI (e.g., NNNNTGNNNN) to avoid homopolymers that may introduce errors due to polymerase slippage or low accuracy of Nanopore sequencing in these sequences. Several studies have also pointed out that both Illumina and PacBio are prone to errors in such regions [20, 21]. The structured UMI design also serves as a quality control in the UMI analysis. The 5′ universal primer sequence is used to uniformly amplify all UMI tagged DNA molecules. It is designed to avoid non-specific priming in the target genome.

### UMI labeling

The primers used in this study are shown in Additional file 1: Table S3. Genomic DNA was extracted using the Qiagen DNeasy Blood & Tissue Kit. The concentration was determined using a Qubit 4 Fluorometer (ThermoFisher). The UMI labeling step was done by one round of primer extension with a high-fidelity DNA polymerase. The reaction setup was similar to a standard PCR reaction, but with only one UMI primer. The UMI labeling reaction was set up as follows: 50 ng DNA, 1 μM UMI primer, 12.5 μl 2X Platinum™ SuperFi™ PCR Master Mix, and H_2_O in a total volume of 25 μl. The UMI labeling was performed on a thermocycler with a ramp rate of 1 °C per second using the following program: 98 °C 1 min, 70 °C 5 s, 69 °C 5 s, 68 °C 5 s, 67 °C 5 s, 66 °C 5 s, 65 °C 5 s, 72 °C (5 min for the 7 kb targets, 10 s for the 168 bp target), 4 °C hold. After UMI labeling DNA was purified by AMPure XP beads, followed by PCR amplification using the universal primer and the gene-specific reverse primer. This amplification generated enough UMI-labeled DNA for downstream sequencing. In addition to one-ended labeling, two-ended UMI labeling can also be achieved by performing an additional UMI-labeling step with a reverse primer tagged with a UMI (Supplementary Fig. 2a). Two-ended UMI labeling could increase analyzable reads and provides extra benefit in accuracy. However, we found that due to the fact that UMI labeling is limited by primer efficiency, one-ended labeling will cover more molecules. Additional UMI-labeling and purification steps resulted in higher loss of DNA of interest. Since the procedure of one-ended labeling was simple and efficient, we used one-end UMI labeling for all experiments in this study.

### Library preparation and sequencing

For Nanopore sequencing, library preparations were done using the ligation sequencing kit (Cat# SQK-LSK109, Oxford Nanopore Technologies). The sequencing runs were performed on an Oxford Nanopore MinION sequencer using R9.4.1 flow cells. Base calling of Nanopore reads was done using the official tool termed Guppy (v3.2.1). For PacBio sequencing, library preparations were done using the Sequel Sequencing Kit 3.0. The sequencing runs were performed by the BIOPIC core facility at Peking University (Beijing, China) on a PacBio Sequel using Sequel SMRT Cell 1 M v3. HiFi Reads were generated by the official tool termed ccs (v3.4.1). All procedures were preformed according to the manufacturer’s protocols. For Illumina sequencing, library preparations were performed using the NEBNext Ultra II DNA Library Prep Kit for Illumina. An unrelated RNA library prepared using the same kit was pooled to increase the complexity of final library. The sequencing of paired-end 150 bp reads was done on an Illumina Miniseq.

### Data processing

VAULT was developed for data analysis. Most of the codes were written in Python 3.7, while some modules were written in Bash. In general, VAULT uses several published algorithms for UMI extraction, alignment, and variant calling. By default, it utilizes cutadapt [22], minimap2 [23], samtools [24], and sniffles [25]. The whole analysis can be done with one command. In brief, Nanopore reads are trimmed to remove adapter sequences and then aligned to the reference gene for extraction of mappable reads. Cutadapt is used to extract UMI sequence, followed by counting of the occurrence of each UMI, which reflects the number of reads in each UMI group. If a structured UMI (NNNNTGNNNN) is used in the experiment, the program will also check the UMI structure. Next, based on a user-defined threshold of minimum reads per UMI group, the program bins reads for eligible UMIs. The grouped reads will be subjected to minimap2 for alignment, followed by SNP calling by samtools and SV calling by sniffles. After finishing all variant calling, a final data cleanup is performed to combine individual variant call files (VCF) together and filter the VCF based on variant quality, depth, and VAF. The number of reads in UMI groups and the corresponding UMI sequence will be written in the ID field of the VCF. Individual folders named by the UMI sequence will be saved to contain the alignment summaries and BAM files of every UMI group. VAULT supports both long-read data and single-end/paired-end short-read data. The data analysis pipeline employs parallel computing for each UMI group, which avoids crosstalk during data analysis and accelerates the process. A typical analysis of 2.5 million long reads will take around 4 h on a 32-core workstation. The somatic SNV load is calculated as:

$$ \mathrm{Number}\ \mathrm{of}\ \mathrm{somatic}\ \mathrm{SNVs}/\left[\mathrm{number}\ \mathrm{of}\ \mathrm{UMI}\ \mathrm{groups}\times \mathrm{surveyed}\ \mathrm{region}\ \mathrm{length}\right]. $$

The primer length was excluded in the surveyed region length. For a rare mutation with known estimated frequency such as 1:100, we estimated that to observe at least one mutant UMI group 90% of the time, the minimal number of UMI group is 229 (*p*[≧ 1 observation] = 1-*p*[0 observation] = 1–0.99n, if *p*[≧1 observation] > 90%, then *n* ≧ 229). The subsampling of reads was performed using *seqtk subsample*. The analysis of *PANX1*-related sequencing data was done using VAULT with the *--group_filter* option to remove low-confidence UMI groups (details in VAULT manual). The SNV annotation was performed using SnpEff [26] v4.3 with the hg38 database.

## Supplementary information

**Additional file 1: Figs. S1-S8.** Supplementary Figures. **Tables S1-S3.** Supplementary Tables.

**Additional file 2.** Review history.

## Data Availability

VAULT and sample data in this study are accessible at GitHub under the GPL-3.0 open source license [27]. The version of VAULT used in this study is deposited on Zenodo with the doi: 10.5281/zenodo.3977107 [28]. Raw sequencing data are available in the SRA database (accession ID PRJNA606194), which are accessible with the following link: https://www.ncbi.nlm.nih.gov/bioproject/PRJNA606194 [29].
