# Supplementary material for: Long-read individual-molecule sequencing reveals CRISPR-induced genetic heterogeneity in human ESCs
Source: Genome Biol. 2020 Aug 24;21:213. doi: 10.1186/s13059-020-02143-8 (PMC7444080; doi:10.1186/s13059-020-02143-8)

Additional file 1 for

**Long-read individual-molecule sequencing reveals CRISPR-induced genetic heterogeneity in human ESCs**

Chongwei Bi, Lin Wang, Baolei Yuan, Xuan Zhou, Yu Li, Sheng Wang, Yuhong Pang, Xin Gao, Yanyi Huang, Mo Li

Correspondence to: yanyi@pku.edu.cn (YH), [mo.li@kaust.edu.sa](mailto:mo.li@kaust.edu.sa) (ML)

**This PDF file includes:**

Figs. S1 to S8

Tables S1 to S3

Fig S1, Advantages of IDMseq and VAULT as compared to current methods

A given population of cells (symbolized by the blue shape) may contain different alleles of a target locus, which accounts for a small proportion of the pool of genomic DNA. The first step of targeted molecular consensus sequencing is labeling of the variant alleles with UMI. Ligation-based and PCR-directed UMI labeling are two of the most widely used methods. Ligation-based UMI labeling will label irrelevant regions and the low efficiency of ligation will also omit a proportion of target alleles (greyed out in the middle left panel). PCR-directed UMI labeling is highly efficient but will result in UMI clashes (one original molecule labeled with multiple UMIs, leading to false UMI groups, middle right panel). IDMseq is the only method with high labeling efficiency and can faithfully retain the allele information (variants and frequency). After UMI labeling, the DNA with UMIs are amplified for sequencing in appropriated platforms (Illumina, Nanopore or PacBio). In the data analysis step, the algorithm needs to identify reads with the same UMI and use these to get the consensus sequence of the allele. This step is currently done with read-clustering algorithms that work well for fixed-length reads of short-read sequencing (e.g. Illumina). However, this strategy could miss reads with complex changes such as those uncovered by long-read sequencing, which prevents detection of deletions, insertions and complex structural variants (lower left panel). VAULT performs a BLAST-like strategy to locate UMI sequence in reads regardless of length and structure. VAULT analysis thus preserves the sequence information of all types of alleles and their frequency (lower middle and right).

Fig S2, Overview of IDMseq and VAULT

**(a)** Schematic representation of UMI labeling. UMI primers are used to label individual DNA molecules with unique UMIs (one molecule is labeled with one UMI). It contains a 3’ gene-specific sequence, a UMI sequence, and a 5’ universal primer sequence. The 3’ gene-specific sequence is selected for its high specificity to the target gene. The middle UMI sequence consists of multiple random bases (denoted by Ns). The 5’ universal primer sequence is used to uniformly amplify all UMI-tagged DNA molecules. IDMseq is different from other UMI-based methods in that barcoding is achieved by a single round of primer extension rather than multiple cycles of PCR as commonly practiced^1,2^. The forward UMI primers will be removed before amplification by the universal primers. For two-ended labeling, an additional round of primer extension with reverse UMI primers will be done after removing forward UMI primers. The UMI-labeled DNA will be further amplified by universal primers before sequencing.

**(b)** Pipeline of VAULT analysis. During data pre-processing, raw reads were filtered and mappable reads were extracted. After that, VAULT applies a BLAST-like strategy to locate UMI sequence in reads by searching for the known sequences of the universal primer and gene-specific forward primer. After that, VAULT bins reads according to UMI. The last steps of VAULT are variant calling for both SNVs and large SVs and report generation.

Fig S3, Generation of isogenic knock-in hESCs using CRISPR-Cas9

**(a)** Schematic representation of the experimental design. Cas9 RNP and ssODN were electroporated to H1 ESCs to generate homozygous G>A single-base substitution in the EPOR gene.

**(b)** Schematic of the Cas9 target site and the NcoI restriction site. The gel image shows restriction enzyme digestion assay used to identify the knock-in hESC clones. Wild-type *EPOR* gene contains a NcoI site and thereby can be digested. The Knock-in allele will lose the NcoI site and cannot be digested.

**(c)** Sanger sequencing results confirming the knock-in SNV. The homozygous SNV is indicated by the black arrow.

Fig S4, IDMseq for detection of the knock-in SNV

**(a)** Two examples of Integrative Genomics Viewer (IGV) tracks of UMI groups in which the spike-in SNV in the 1:100 population was identified by Nanopore sequencing in conjunction with IDMseq and VAULT. The knock-in SNV is indicated by the red triangle in the diagram of the EPOR gene on top, and also shown as red “T” base in the alignment map. The gray bars show read coverage. The ten colored bars on the left side of the coverage plot represent the UMI sequence for the UMI group. Individual Nanopore reads within the group are shown under the coverage plot.

**(b)** Aligned read length vs. percent identity plot using kernel density estimation for Nanopore sequencing of the 1:10,000 population, Illumina sequencing of the 1:10,000 population, PacBio sequencing of the 1:1,000 population.

**(c)** The read number distribution for UMI groups in Nanopore, Illumina and PacBio sequencing runs. The distribution is shown as read number per UMI group vs. UMI group count. For each sequencing run, it plots the distribution of all UMI groups and variant UMI groups.

Fig S5, Analysis of CRISPR-Cas9 on-target mutagenesis

**(a)** Aligned read length vs. percent identity plot using kernel density estimation of Nanopore sequencing data of Pan1 Cas9 editing experiment.

**(b)** similar to **(a)** but for Pan3 Cas9 editing experiment.

**(c)** Individual alleles from Sanger sequencing of single-cell derived hESC clones after Cas9-directed mutagenesis in exon 1 of *PANX1*. Green letters indicate the gRNA sequence and the cleavage site is indicated by a dotted line. Red texts indicate insertion or deletion events.

**(d)** similar to **(c)** but with the Pan3 sgRNA.

**(e)** IGV tracks of the UMI groups that shared the same deletion as alleles detected by Sanger sequencing of hESC clones (highlighted by a blue box in **(c)** and matching symbols). The red dotted line shows where Cas9 cuts.

**(f)** similar to **(e)** but with the Pan3 sgRNA.

**(g)** Distribution of SNVs detected by IDMseq and VAULT in Pan3 edited hESCs. Somatic SNVs are shown in green, while the cell-line specific SNVs are shown in red. Somatic SNVs cannot be detected if variant calling is done en masse without UMI analysis (see the coverage track). Cell-line specific SNVs are detected in ensemble analysis (see colored lines in the coverage track) and most of them have been reported as common SNPs in dbSNP-141 database (Common SNPs track). The Cas9 cut site is indicated by the red triangle.

Fig S6, Analysis of variants in PANX1 edited hESCs

**(a)** The frequency of different size deletions, insertions and inversions detected in Pan3-edited hESCs. Certain deletions and insertions occur at disproportionally high frequencies. For example, a 4238 bp deletion was found in 27 UMI groups, which indicates a possible hotspot of Cas9-induced large deletion.

**(b, c)** Frequency distribution of the variant allele fraction of SNVs detected by IDMseq in Nanopore sequencing of the *PANX1* locus in Pan1-edited hESCs **(c)**, and Nanopore sequencing of the *PANX1* locus in Pan3-edited hESCs **(c)**.

**(d)** Analysis of somatic mutations detected in Pan3-edited hESCs based on functional annotation and base change. The majority of base changes are G to A and C to T.

Fig S7, Analysis of variants in wild-type H1 hESCs

**(a)** Distribution of SNVs detected by IDMseq and VAULT in Pan1 control sequencing. Somatic SNVs are shown in green, while the cell-line specific SNVs are shown in red. Somatic SNVs cannot be detected if variant calling is done en masse without UMI analysis (see the coverage track). Cell-line specific SNVs are detected in ensemble analysis (see colored lines in the coverage track) and most of them have been reported as common SNPs in dbSNP-141 database (Common SNPs track).

**(b)** similar to **(a)** but for Pan3 control sequencing.

**(c)** Analysis of somatic mutations detected in Pan1 control sequencing based on functional annotation, base change and allele frequency distribution. The majority of base changes are G to A and C to T.

**(d)** similar to **(c)** but for Pan3 control sequencing.

Fig S8, Subsampling analysis of the sequencing data of Cas9-edited hESCs

**(a)** Boxplots showing the distribution of somatic SNV numbers (y-axis) detected in the >100 subsamplings of the Pan1 edited sample at the subsampling read depth indicated on the x-axis. The red dots inside the boxes indicate the mean numbers of detected somatic SNVs, and the thick black lines represent the median numbers. Potential outliers were shown as black dots outside the boxes. The lower and upper boundaries of the box represent the 25^th^ and 75^th^ percentiles, respectively.

**(b)** Boxplots showing the distribution of SV numbers detected in the >100 subsamplings of the Pan1 edited sample at the subsampling read depth indicated on the x-axis. The red dots inside the boxes indicate the mean numbers of detected SVs, and the thick black lines represent the median numbers. The lower and upper boundaries of the box represent the 25^th^ and 75^th^ percentiles, respectively.

**(c)** similar to **(a)** except for Pan3 subsampling analysis.

**(d)** similar to **(b)** except for Pan3 subsampling analysis.

Table S1: Subsampling analysis of *PANX1* sequencing

The color coding indicates the number of subsampled reads and the number of reads in the WT experiment that the subsampling is designed to match.

Table S2: IDMseq vs. nCATS

Table S3: Primers used in this study


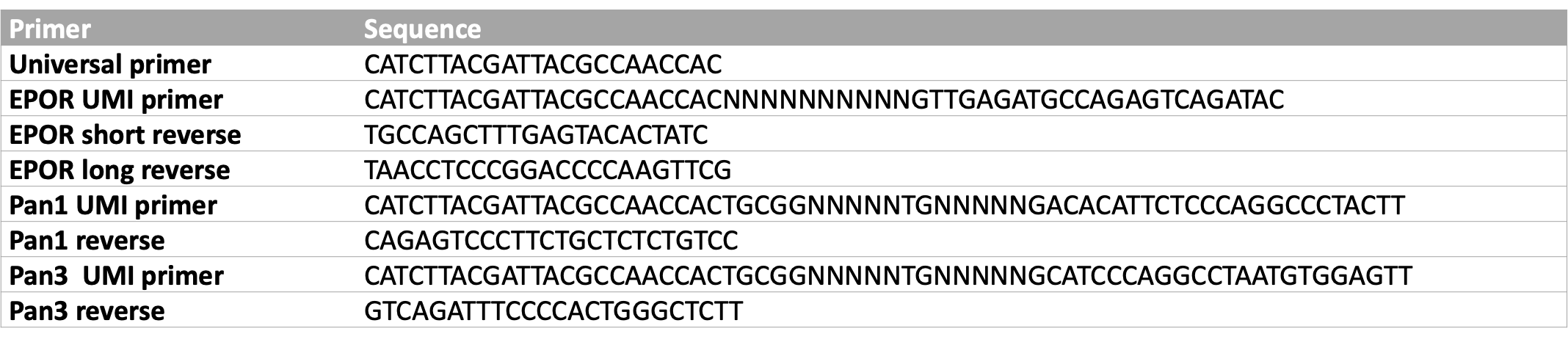

Supplement: Supplementary file 1 — Additional file 1: Figs. S1-S8. Supplementary Figures. Tables S1-S3. Supplementary Tables. [file 13059_2020_2143_MOESM1_ESM.docx]
