# Supplementary material for: Long-read individual-molecule sequencing reveals CRISPR-induced genetic heterogeneity in human ESCs
Source: Genome Biol. 2020 Aug 24;21:213. doi: 10.1186/s13059-020-02143-8 (PMC7444080; doi:10.1186/s13059-020-02143-8)
Supplement: Supplementary file 2 — Additional file 2. Review history. [file 13059_2020_2143_MOESM2_ESM.docx]

Review History

**First round of review**

**Reviewer 1**

**Were you able to assess all statistics in the manuscript, including the appropriateness of statistical tests used?**

No

**Were you able to directly test the methods?**

No

**Comments to author:**

The authors present a method for targeted long-read sequencing using UMIs to increase sensitivity for detecting rare structural variants. They used controlled genome mixes to validate the high sensitivity of the method, and then quantitatively surveyed large structural variants introduced by the CRISPR-Cas9 system in human ESCs. Overall, this method is well designed and potentially useful in discovering rare SVs around loci of interest.

I have some questions. Please correct me if I misunderstood some points.

1. It is known that the CRISPR-Cas9 system introduces large SVs, and has been prooved in mESCs as pointed out by the authors. What's the value of proving this fact again in hESCs? Besides, large SVs represent a significant portion of the Cas9 mutagenesis effect, what's the benefit to measure this effect with a highly sensitive method?

2. The authors tested the method with different settings in the EPOR case. In these settings, the known mutation was observed in only 1, 2, 4, and 5 UMI groups. In those experiments with only 1 or 2 UMI groups, if a few positive reads were missed, the experiments might be failing in observing the mutation. It is not convincing to call this a "reliable detection" (page 5, line 12). Can the authors at least calculate reads amount required to identify the known mutation in at least one UMI group with a reasonable confidence?

3. The UMI labeling rates calculated by (reads with UMI / total reads) are relatively low, according to Table 1 (about 20-40% for the Nanopore platform). As the cost of long-read sequencing is high, the low labeling rate means a considerable waste of sequencing capacity. What's the reason? Is there any way to improve that?

4. What is the size (reads number) of those SV/SNV positive UMI groups? Long reads technologies have high error rates that need to be corrected by multiple reads. Is the size of these positive groups large enough to correct the sequencing errors? This is particularly important to detect SNVs accurately.

5. Other than indels, did authors found any inversions? This might be difficult for the current algorithms.

6. What are the major differences between IDMseq and PCR-directed UMI labeling? The number of PCR cycles?

7. It will be nice to see a fair comparison with nCATS in the main text. That method could target multiple loci simultaneously, while the authors didn't mention this point in the letter.

8. Detecting large structural variants using long-reads technologies has been improved rapidly in recent years. However, the citations of this paper are kind of old, with only one published in 2019.

**Reviewer 2**

**Were you able to assess all statistics in the manuscript, including the appropriateness of statistical tests used?**

Yes: The validation of the IDMseq approach lacks statistical analysis and biology replicates to benchmark the SVs identified are real somatic mutations or not just technical noises. The bioinformatics pipeline lacks statistical evaluation.

**Were you able to directly test the methods?**

No

**Comments to author:**

The authors developed a UMI based approach to label target DNAs to quantitative characterize the DNA sequences in haplotype resolution using short- or long-read sequences. The short read UMI approaches have been widely used, but these approaches can't detect large structure variations because of the length limitation. The authors applied the IDMseq approach to investigate the large SVs induced by CRISPR-Cas9 approaches, which is a significant biology question.  The manuscript is well written and presented. Both the long read based approach and the structure variations identified in CRISPR-Cas9 editing are significant and would be valuable to the community. However, the validation of the IDMseq approach lacks statistical analysis and biology replicates to benchmark the SVs identified are real somatic mutations or not just technical noises. In addition, the study in CRISPR-Cas9 editing misses the WT cell control to distinguish real Cas9 induced SVs from somatic SVs.

a) Major comments:
1. The statistical evaluation/description of the VAULT analysis is missed. For example, how is the threshold for the minimum number of reads per UMI determined? The authors mentioned: "pre-set threshold of a minimum of 5 reads per UMI group." Are 5 reads per UMI used for all samples? It should be related to the number of reads generated. What is the mean/median value of the number of reads per UMI? What is the distribution of the number of reads per UMI?
2. What is the error rate of the UMI barcode classification? It is clearly in Fig1d, there are multiple reads that are not including the large deletion. And in Fig2C, it seems 10~30 percent of reads doesn't have the deletion while they share identical UMIs. How does this affect the SNV and SV calling? For example, what is the allelic frequency distribution of all the SV/SNVs in one UMI group?
3. As shown in Table1, the somatic SNV per megabase vary from 3.8 to 14.4. Why are there such big differences? I noticed that the number is somehow correlated with the length of the amplicon sizes and the number of reads with UMIs. It seems longer amplicon sizes (and more reads with UMIs), the more SNVs per megabase are detected. This suggests some of the SNVs detected are caused by technical problems/noises during PCR or detection pipeline of SNVs rather than real somatic SNVs.
4. In the analysis of PANX1 edited samples, a control sequencing of WT cells is missed. So it is not clear what is the threshold to decide one SNV/SV is a Cas9-induced deletion/insertion or somatic mutation? The authors should compare to the sequences of WT cells to call out the real Cas9-induced SNVs/SVs.
5. Page 5, line 43 & Page 6 line 5. "For example, 56 (28.0%) UMI groups have the same 5,494-bp deletion and 18 (9.0%) UMI groups have the same 4,715-bp deletion." This is interesting that the deletions have a different number of UMIs. The authors conclude  "first quantitative evidence that the repair outcome of Cas9 cutting is not random and there are likely hotspots for Cas9-induced large deletions or insertions." For this conclusion, biology replicates are needed to statistically rule out the possibility of just the technical noise of random numbers. On the other side, can the authors rule out the possibility that certain deletion may cause growth differences?
6. Page 4, the comparison between Illumina and Nanopore long reads for the 168 bp 1:10,000 mixed population is missed. The authors suggest the 10 non-knocked-in SNVs detected in Nanopore sequencing are somatic mutations. Are they also detected in Illumina sequencing? They should be as there are more reads in Illumina sequencing. In addition, some of the numbers do not match very well: for example, there are on average 32 (494009/15598) reads per UMI for Nanopore sequencing, and 55 (7236007/132341) reads per UMI for Illumina sequencing. How come are there only 6 reads for knocked-in SNV UMIs in Fig1b?

b) Minor comments
1. Page 5, line 3. "Functional annotation of the SNVs showed that 17 of 192 caused an amino acid change." The number (192) doesn't fit what is shown in Fig. 1d.
2. The description of how the number of somatic mutations is calculated is missed in the method.
3. Page 5, line 40. "After SV calling and filtering out lowly supported SVs (see methods)," How SV is called and filtered is not clearly described in methods.
4. Fig 2e, there is one SNP showing high frequency (second line (red/green) from the left in the coverage panel), but the coverage panel on top missed it.

**Authors Response**

**Point-by-point responses to the reviewers’ comments:**

Reviewer #1:

The authors present a method for targeted long-read sequencing using UMIs to increase sensitivity for detecting rare structural variants. They used controlled genome mixes to validate the high sensitivity of the method, and then quantitatively surveyed large structural variants introduced by the CRISPR-Cas9 system in human ESCs. Overall, this method is well designed and potentially useful in discovering rare SVs around loci of interest.

I have some questions. Please correct me if I misunderstood some points.

1. It is known that the CRISPR-Cas9 system introduces large SVs, and has been proved in mESCs as pointed out by the authors. What's the value of proving this fact again in hESCs? Besides, large SVs represent a significant portion of the Cas9 mutagenesis effect, what's the benefit to measure this effect with a highly sensitive method?

*Reply: We thank the reviewer for raising the question. To the best of our knowledge, it is unclear if different cell lines behave the same following Cas9-based gene editing. Human ESCs (hESCs) is considered to be in the primed pluripotency state, which differ in chromatin state and bioenergetics from the naïve state of mESCs. Therefore, one cannot formally rule out possible differences in the propensity of Cas9-induced large SVs between hESCs and mESCs. Besides, hESCs are widely used in both basic research and clinical applications. We therefore believe it is important to report the DNA repair outcome in hESCs after Cas9-based editing in an unbiased fashion.

Previous studies showed large SVs are a part of the Cas9 mutagenesis effects, but the mutagenesis profiles such as types and frequency of the large SVs are not well characterized mainly due to the lack of unbiased and sensitive quantification methods. For example, those studies relied on PCR amplification of the target regions for bulk sequencing, which could potentially over-estimate large deletion events due to PCR bias towards short amplicons. On the other hand, bulk sequencing of PCR amplicon obscures low-frequency events, thus potentially underestimating the diversity of SVs. Our individual DNA molecule sequencing method solves these problems by offering both high sensitivity and reliable quantification at the same time. What’s more, our method revealed that a significant number of SVs (up to 87%) are reoccurring deletions and insertions. This phenomenon is only discovered by IDMseq.

We’d also like to add that previous works did not report accumulation of SNV after Cas9 editing. Again, this is due to the lack of unbiased and sensitive quantification methods. Here in the revised manuscript, we reported for the first time quantitative analyses of SNVs before and after Cas9 editing, and showed a significant increase in somatic SNVs following Cas9 editing.*

2. The authors tested the method with different settings in the EPOR case. In these settings, the known mutation was observed in only 1, 2, 4, and 5 UMI groups. In those experiments with only 1 or 2 UMI groups, if a few positive reads were missed, the experiments might be failing in observing the mutation. It is not convincing to call this a "reliable detection" (page 5, line 12). Can the authors at least calculate reads amount required to identify the known mutation in at least one UMI group with a reasonable confidence?

*Reply: We thank the reviewer for raising the question. It is true that those 1 or 2 UMI groups might be overlooked if a few positive reads were missing. This possibility is mainly due to an insufficient sequencing depth that is unable to support the discovery of enough UMI groups. For a rare mutation with known estimated frequency such as 1:100, we estimated that to observe at least one mutant UMI group 90% of the time, the minimal number of UMI group is 229 (p[≧1 observation]=1-p[0 observation]=1-0.99n, if p[≧1 observation]>90%, then n≧ 229). In the EPOR case, we obtained 284 UMI groups, which is estimated to produce a positive observation 94% of the time. Our actual data matched well with this expectation, which indicated that our method is effective and reliable. The Oxford Nanopore MinION, being a flexible technology platform, allows one to monitor sequencing output in real-time. One can decide to stop the sequencing run when enough coverage is reached, or to do catch-up runs if more reads are desired. For example, if we stopped the sequencing at 170k reads, we would expect to identify ~ 2840 UMI groups and 20 UMI groups with mutations.

As shown in the manuscript, the number of reads required to identify the known mutation is dependent on the experiment setting, e.g. sequencing platform, amplicon size, primer design, etc. Higher raw read accuracy, shorter amplicon size and efficient UMI-labeling primers tend to facilitate identification of UMI groups.

We have added the discussion of reads amount required to identify the known mutation in at least one UMI group with a reasonable confidence in the methods section on Page 10 of the revised manuscript.*

3. The UMI labeling rates calculated by (reads with UMI / total reads) are relatively low, according to Table 1 (about 20-40% for the Nanopore platform). As the cost of long-read sequencing is high, the low labeling rate means a considerable waste of sequencing capacity. What's the reason? Is there any way to improve that?

*Reply: There are at least two reasons for the low labeling rate. First, both strands of the DNA duplex have an equal chance of being sequenced from 5’ to 3’. If the UMI resides on the 3’ of the strand, and the nanopore terminates the sequencing before reaching the 3’ end, this read won’t contain any UMI sequence. Such sub-full length reads are generated stochastically during nanopore sequencing. Future improvement in the chemistry of the nanopores may solve this issue. Second, sequencing errors in the UMI region contribute to the failure to identify UMI in some reads. As shown in Table 1, in contrast to Nanopore sequencing (raw read accuracy ~90%), the highly accurate Illumina sequencing (raw read accuracy 99.9%) offers 96.63% of UMI-containing reads. Since we utilized a BLAST-like strategy for UMI identification, the UMI regions with too many errors will not pass the error tolerance threshold.

To improve the number of usable reads, VAULT offers a parameter [-e] to define the error tolerance threshold. The default value is set based on the average sequencing error rate. Increasing this value will yield more reads with UMI. However, the user should ensure that relaxing this parameter doesn’t lead to creation of false UMI groups. Another parameter [-u] can also be used to adjust threshold of UMI identification. It asks for known DNA sequences flanking the UMI, and the BLAST algorithm will search for these sequences to identify UMIs. User can shorten the length requirement of the flanking sequences to potentially obtain more reads with UMIs. Independent of the computational remedies described above, Oxford Nanopore Technologies have release new pore chemistry called R10 that significantly increase read accuracy. VAULT can take full advantage of such improvements in sequencing chemistry and make the method more cost-efficient.

We have added the discussion of increasing reads with UMI in the manual of VAULT (https://www.dropbox.com/sh/er2etuoz4cniftl/AADlCajM0Wnjlx8HvsIeW-t5a?dl=0).*

4. What is the size (reads number) of those SV/SNV positive UMI groups? Long reads technologies have high error rates that need to be corrected by multiple reads. Is the size of these positive groups large enough to correct the sequencing errors? This is particularly important to detect SNVs accurately.

*Reply: We have added the size (read number) distribution of UMI groups on Page 4 and Figure S4c in the revised manuscript.

Currently, long read sequencing technologies such as Nanopore and PacBio have significantly increased their read accuracy through better sequencing chemistry and base-calling algorithm. As shown in the published work (PMID: 31213642) and our in-house analysis, a read coverage of 5 can provide confident SNV and SV calling result. VAULT provides a parameter [-T] to define the minimum read number requirement of UMI groups. Based on our data, we set the default value to 5. In addition, VAULT contains a stringent variant filter to screen detected variants based on variant quality, depth, supported fraction percentage, etc. It also contains a filter [--group_filter] to filter out low confidence UMI groups. All of these features of VAULT support a reliable variant calling.*

5. Other than indels, did authors found any inversions? This might be difficult for the current algorithms.

*Reply: As we stated on page 6, line 5 in the original manuscript, there are 12 inversions found in Pan3 sequencing with length range from 296 bp to 2750 bp. The SV calling algorithm (Sniffles, PMID: 29713083) has been shown to be able to detect inversions.*

6. What are the major differences between IDMseq and PCR-directed UMI labeling? The number of PCR cycles?

*Reply: The traditional PCR-directed UMI labeling uses both the forward and reverse UMI primers to amplify the target in several cycles of PCR. This results in a single molecule being labeled with multiple UMIs. In IDMseq, we use only one primer (UMI primer) to do one round of primer extension. We also performed a gradient annealing (in method, Page 9, line 2) to increase the efficiency of primer annealing and extension. The use of one primer in a single round of primer extension and the thermocycler condition differentiate IDMseq from PCR-directed UMI labeling, and allow IDMseq to achieve true unique individual molecule labeling.*

7. It will be nice to see a fair comparison with nCATS in the main text. That method could target multiple loci simultaneously, while the authors didn't mention this point in the letter.

*Reply: This is a valuable suggestion. We have added the comparison on Page 6 line 34, and Supplementary Table 2 in the revised manuscript.*

8. Detecting large structural variants using long-reads technologies has been improved rapidly in recent years. However, the citations of this paper are kind of old, with only one published in 2019.

*Reply: We cited more recent publication on structural variation calling (Page 3, line 14) and targeted Nanopore sequencing (nCATS) (Page 6, line 35) in the revised manuscript.*

Reviewer #2: The authors developed a UMI based approach to label target DNAs to quantitative characterize the DNA sequences in haplotype resolution using short- or long-read sequences. The short read UMI approaches have been widely used, but these approaches can't detect large structure variations because of the length limitation. The authors applied the IDMseq approach to investigate the large SVs induced by CRISPR-Cas9 approaches, which is a significant biology question. The manuscript is well written and presented. Both the long read based approach and the structure variations identified in CRISPR-Cas9 editing are significant and would be valuable to the community. However, the validation of the IDMseq approach lacks statistical analysis and biology replicates to benchmark the SVs identified are real somatic mutations or not just technical noises. In addition, the study in CRISPR-Cas9 editing misses the WT cell control to distinguish real Cas9 induced SVs from somatic SVs.

a) Major comments:
1. The statistical evaluation/description of the VAULT analysis is missed. For example, how is the threshold for the minimum number of reads per UMI determined? The authors mentioned: "pre-set threshold of a minimum of 5 reads per UMI group." Are 5 reads per UMI used for all samples? It should be related to the number of reads generated. What is the mean/median value of the number of reads per UMI? What is the distribution of the number of reads per UMI?

*Reply: We thank the reviewer for raising these questions. VAULT provides a parameter [-T] to define the minimum read number requirement of UMI groups. We agree that this threshold may need to be adjusted in different scenario. The default threshold of 5 reads per UMI group is determined based on a published work (PMID: 31213642) and our in-house evaluation of other Nanopore amplicon sequencing data (no UMIs, unpublished). In brief, we subsampled Nanopore reads to simulate various sequencing depth for calling a known mutation, and the results showed that the variant can be faithfully detected at a coverage of 5. As the variant calling algorithm works well for 5 reads, we set this value for all samples to maximize the number UMI groups. Using this setting, the allele frequency of the introduced mutation detected by VAULT agrees well with the ground truth allele frequency in the cell population, which further suggests that the threshold of 5 reads per UMI works well under our experimental settings.

We have added the distribution and median value of the number of reads per UMI group in Figure S4c and Table 1 in the revised manuscript.*

2. What is the error rate of the UMI barcode classification? It is clearly in Fig1d, there are multiple reads that are not including the large deletion. And in Fig2C, it seems 10~30 percent of reads doesn't have the deletion while they share identical UMIs. How does this affect the SNV and SV calling? For example, what is the allelic frequency distribution of all the SV/SNVs in one UMI group?

*Reply: The error tolerance rate of BLAST-based UMI barcode classification is set at 0.1 for Nanopore and PacBio data, and 0.05 for Illumina data. The value is set based on the error rate of different sequencing runs. Raw read errors in the UMI will fortuitously group wrong reads in the UMI group. This is especially significant for sequencing technologies with high raw-read error rate. To avoid wrong variant calling in such groups, we applied a stringent variant filter to screen detected variants based on variant quality, depth, supported fraction percentage (allelic frequency), etc. The allele frequency cutoff was 66.7%, which means all of the reported SVs/SNVs have an allelic frequency greater than 66.7%. Thus, this variant filter ensures that the low percentage reads (presumably wrongly grouped due to errors in UMI) pointed out by the reviewer will not affect SNV and SV calling.*

3. As shown in Table1, the somatic SNV per megabase vary from 3.8 to 14.4. Why are there such big differences? I noticed that the number is somehow correlated with the length of the amplicon sizes and the number of reads with UMIs. It seems longer amplicon sizes (and more reads with UMIs), the more SNVs per megabase are detected. This suggests some of the SNVs detected are caused by technical problems/noises during PCR or detection pipeline of SNVs rather than real somatic SNVs.

*Reply: We thank the reviewer for this insightful question. Upon close examination, we identified the source of the discrepancy lies in the formula of somatic SNV load calculation, which was:
number of somatic SNVs / [number of UMI groups × amplicon length].

The use of amplicon length in the formula is not accurate, because it contains the primer sequences that are invariant. Although the length of primers is minuet compared to a long amplicon (e.g., 6.8 kb), it represents a significant portion of a short amplicon (e.g. 168 bp). After correcting for this, the somatic mutation load is consistent in EPOR experiments. We have updated Table 1 and the text (Page 4 line 22 and 33, Page 5 line 6) with the correct numbers. The formula for somatic SNV load calculation has been added in the method section. We have also included new data of the PANX1 amplicons in wild-type H1 hESCs (also see the answer to question 4 below). Overall, the correlation between length of the amplicon and somatic SNV per megabase no longer exist. Furthermore, our new data showed that the somatic SNV load in wild-type H1 hESCs before Cas9 editing is ~ 3-fold lower than that in Cas9-edited H1 hESCs. Together these observations suggest that most somatic SNVs are true variants.*

4. In the analysis of PANX1 edited samples, a control sequencing of WT cells is missed. So it is not clear what is the threshold to decide one SNV/SV is a Cas9-induced deletion/insertion or somatic mutation? The authors should compare to the sequences of WT cells to call out the real Cas9-induced SNVs/SVs.

*Reply: We thank the reviewer for suggesting this experiment. We performed IDMseq and VAULT analysis of wild-type H1 hESCs that were cultured in parallel as those used in Cas9 editing experiments. The results show that no SV was detected in wild-type WT cells, and that the somatic SNV load in wild-type H1 hESCs before Cas9 editing is ~ 3-fold lower than that in Cas9-edited H1 hESCs. These data were presented on Page 5 and Figure S7 in the revised manuscript.*

5. Page 5, line 43 & Page 6 line 5. "For example, 56 (28.0%) UMI groups have the same 5,494-bp deletion and 18 (9.0%) UMI groups have the same 4,715-bp deletion." This is interesting that the deletions have a different number of UMIs. The authors conclude "first quantitative evidence that the repair outcome of Cas9 cutting is not random and there are likely hotspots for Cas9-induced large deletions or insertions." For this conclusion, biology replicates are needed to statistically rule out the possibility of just the technical noise of random numbers. On the other side, can the authors rule out the possibility that certain deletion may cause growth differences?

*Reply: The observation of reoccurring large deletions came from two independent experiments using two independent guide RNAs. Such high frequency or large SVs was not observed in the EPOR locus. Additionally, the new data of wild type H1 hESCs showed no large deletions. Together, these data showed our method is consistent and specific, and the reoccurring large deletions are not likely due to technical noise. The reviewer is right that more biological replicates, ideally performed in different loci, will bolster the claim. Unfortunately, our lab is in lockdown and the country is experiencing severe logistical challenges due to covid-19. We don’t have the resources to perform these experiments under current circumstances. We will follow the reviewer’s suggestion to include the importance of more biological replicates in the future and tone down the claim.

The aim of this study is to capture the genetic heterogeneity of Cas9-edited hESCs, so the cells were harvested 48 hours after electroporation of Cas9 RNP. Therefore, any potential growth difference due to the deletions would not have time to manifest in the data. We hope that our data of reoccurring large deletions will raise the interest to study the impact of different variants on cell survival and growth.*

6. Page 4, the comparison between Illumina and Nanopore long reads for the 168 bp 1:10,000 mixed population is missed. The authors suggest the 10 non-knocked-in SNVs detected in Nanopore sequencing are somatic mutations. Are they also detected in Illumina sequencing? They should be as there are more reads in Illumina sequencing.
In addition, some of the numbers do not match very well: for example, there are on average 32 (494009/15598) reads per UMI for Nanopore sequencing, and 55 (7236007/132341) reads per UMI for Illumina sequencing. How come are there only 6 reads for knocked-in SNV UMIs in Fig1b?

*Reply: We add the comparison of Nanopore and Illumina 168 bp amplicon sequencing on Page 4, line 30 as follow:
“The Illumina sequencing detected 85 somatic SNVs in which 7 overlapped with the 10 (70%) detected by IDMseq using Nanopore sequencing.”
Indeed, 7 out of 10 somatic SNVs in Nanopore sequencing are detected by Illumina sequencing.
The number of reads is not distributed evenly among UMI groups. We add the read number distribution of UMI groups in Figure S4c. Please also refer to our answer to Question 1. In Fig1b, we randomly chose UMI groups with a number of reads close to the median number.*

b) Minor comments
1. Page 5, line 3. "Functional annotation of the SNVs showed that 17 of 192 caused an amino acid change." The number (192) doesn't fit what is shown in Fig. 1d.

*Reply: We have double checked the number in Fig. 1d. To the best of our knowledge the histogram shows a total count of 192.*

2. The description of how the number of somatic mutations is calculated is missed in the method.

*Reply: We add the description of how the number of somatic mutations is calculated in methods on Page 10.*

3. Page 5, line 40. "After SV calling and filtering out lowly supported SVs (see methods)," How SV is called and filtered is not clearly described in methods.
 *Reply: We add the description of SV calling and filter in methods. More detail can be found in VAULT (https://www.dropbox.com/sh/er2etuoz4cniftl/AADlCajM0Wnjlx8HvsIeW-t5a?dl=0).*

4. Fig 2e, there is one SNP showing high frequency (second line (red/green) from the left in the coverage panel), but the coverage panel on top missed it.

*Reply: We thank the reviewer for pointing this out. We binned the variants (40 bp per bin) in the histogram on the top to make them clearer. This results that the position of variants on the top may be a little shifted from theirs true position. The lower coverage track from IGV is showing individual bases, so that the top and bottom graphs may not align perfectly. We have added a note in the figure legend on Page 17, line 15.*

**Second round of review**

**Reviewer 1**

The authors have addressed my technical concerns in this revised manuscript, but I am not entirely convinced of the significance of this method. It is known that large SVs represent a significant portion of Cas9 mutagenesis in mESC, the work shown here by identifying rare large SVs in Cas9 edited hESC doesn’t seem to be enough for support the advance of the method. A technique evaluating multiple loci simultaneously or detect SVs at a single-cell level would be much more useful.

**Reviewer 2**

In the revised version of the manuscript, the authors have made significant improvements, including adding the control experiments of wild-type H1 hESCs samples. However, the number of reads of WTs is significantly lower than Cas9 samples, raising the concern of depth biases. In addition, the comparison of short reads to long reads analysis of the same sample, showing a potential high error rate (30%) of VAULT approach.  These concerns need to be addressed before publication.

1) The authors have added the experiment of wild-type H1 hESCs, which significantly improved the manuscript. However, the number of reads and UMI groups are significantly lower in WT comparing to Cas9 editing samples. This raises the concern that the number of somatic SNV/SVs detected in WT is biased duo to less read coverage as the cutoffs used in VAULT approach like >=5 reads per UMI and >66.7% allele frequency cutoff are depth sensitive. The authors should increase the number of reads for WT to a comparable level of Cas9 sequencing to address the concern. I can understand there may be problems caused by COVID-19 for adding the experiment. Thus, at least, the authors need to do permutations by randomly pick up a similar number (200~ 400K) of reads in Cas9 samples to run the VAULT approach hundreds of times, then calculated the mean/median number of somatic SNVs/SVs. This could help to validate the ~ 300% increase of somatic SNVs in Cas9 editing, and rule out the potential sequence depth biases.

2) The authors have added the simple comparison between Illumina and Nanopore long reads for the 168 bp 1:10,000 mixed sample.  “The Illumina sequencing detected 85 somatic SNVs in which 7 overlapped with the 10 (70%) detected by IDMseq using Nanopore sequencing.” Why were there 3 (30%) SNVs are not detected in Illumina sequencing? Does it mean ~30% error rates of the VAULT approach? If this is the case, fair analysis and discussion of how this could affect the conclusions like “quantitative characterization of diverse types of rare variants” are needed.

3) Page 5, line 3. "Functional annotation of the SNVs showed that 17 of 192 caused an amino acid change." The number (192) doesn’t fit what is shown in the table of Fig. 1d.

**Authors Response**

**Point-by-point responses to the reviewers’ comments:**

Reviewer #2: In the revised version of the manuscript, the authors have made significant improvements, including adding the control experiments of wild-type H1 hESCs samples. However, the number of reads of WTs is significantly lower than Cas9 samples, raising the concern of depth biases. In addition, the comparison of short reads to long reads analysis of the same sample, showing a potential high error rate (30%) of VAULT approach. These concerns need to be addressed before publication.

1) The authors have added the experiment of wild-type H1 hESCs, which significantly improved the manuscript. However, the number of reads and UMI groups are significantly lower in WT comparing to Cas9 editing samples. This raises the concern that the number of somatic SNV/SVs detected in WT is biased duo to less read coverage as the cutoffs used in VAULT approach like >=5 reads per UMI and >66.7% allele frequency cutoff are depth sensitive. The authors should increase the number of reads for WT to a comparable level of Cas9 sequencing to address the concern. I can understand there may be problems caused by COVID-19 for adding the experiment. Thus, at least, the authors need to do permutations by randomly pick up a similar number (200~ 400K) of reads in Cas9 samples to run the VAULT approach hundreds of times, then calculated the mean/median number of somatic SNVs/SVs. This could help to validate the ~ 300% increase of somatic SNVs in Cas9 editing, and rule out the potential sequence depth biases.

*Reply: We thank the reviewer for raising this insightful question. In the previous revision we used one full flow cell per sample (WT or Cas9-edited). In this revision, we used another full flow cell to sequence each of the remaining WT libraries. The number of detected UMI groups increased from 1816 to 2810 (155%) for Pan1, and 2213 to 3867 (175%) for Pan3. The number of UMI groups of the WT samples now reached 81% and 53% of that of the Cas9-edited samples for Pan1 and Pan3, respectively. Despite the significant increase in the number of reads and UMI groups, the somatic mutation loads didn’t increase and remained similar as before, as somatic SNVs/Mb changed from 4.0 to 3.8 for Pan1 and 4.8 to 4.1 for Pan3. Furthermore, no SV was detected with higher sequencing depth.

For reasons not immediately clear, Nanopore sequencing of WT cells generated less reads than that of Cas9-edited cells despite using twice as many flow cells. We note that the flow cells used for WT samples were from a different lot than those used for Cas9-edited samples, and were shipped to us during the COVID-19 lockdown. To rule out the possibility that the observed differences in SNVs and SVs were due to sequence depth biases, we matched the sequencing depth of WT and Cas9-edited samples by randomly sampling reads in Cas9-edited samples (methods on Page 11, line 13, Supplementary Table 1, Fig S8). The same WT libraries were sequenced in two batches (Batch 1 and Batch 2, Supplementary Table 1). For Cas9-edited cells, the numbers of subsampled reads were set to match the corresponding raw read numbers of WT Batch 1 (the 5th column of Supplementary Table 1), the numbers of reads with UMI of WT Batch 1 (the 6th column of Supplementary Table 1), or the numbers of reads with UMI of WT Batch 1 + Batch 2. All of the random subsamplings were performed more than 100 times. The results of 623 subsampling experiments showed that our original observation of a significant increase in the number of somatic SNVs and SVs around the cleavage site after Cas9 editing remained robust (Supplementary Table 1). The subsampling experiments showed small variations in the estimated somatic SNV load per Mb and SV frequency, which might be due to the stochasticity of UMI groups with low coverage meeting the stringent quality filter (see methods). The accuracy of allele frequency estimate could be further enhanced by sequencing deeper, as with any high-throughput sequencing method, or by improving base-calling accuracy of Nanopore sequencing, which would in effect increase the number of reads with UMI. Nonetheless, these data from real-world and in silico experiments ruled out any artifact due to sequencing depth biases and validated the increase of somatic SNVs and SVs near the Cas9 cut site following Cas9 editing.

We have updated the figures and tables in the revised manuscript with new analysis results. All PANX1 related sequencing data were analyzed using VAULT with the “--group_filter” option to remove low-confidence UMI groups. The group filter was described in last revision. Briefly, despite consuming more computational resources, the group filter eliminates problematic UMI groups by surveying the consistency among reads within each UMI group, thus providing a conservative yet more confident result.*

2) The authors have added the simple comparison between Illumina and Nanopore long reads for the 168 bp 1:10,000 mixed sample. “The Illumina sequencing detected 85 somatic SNVs in which 7 overlapped with the 10 (70%) detected by IDMseq using Nanopore sequencing.” Why were there 3 (30%) SNVs are not detected in Illumina sequencing? Does it mean ~30% error rates of the VAULT approach? If this is the case, fair analysis and discussion of how this could affect the conclusions like “quantitative characterization of diverse types of rare variants” are needed.

*Reply: We thank the reviewer for raising this question. The 7 overlapping SNVs were identified in multiple UMI groups (between 3 and 11) in Illumina sequencing, while the 3 non-overlapping SNVs were each discovered in one UMI group in Nanopore sequencing. Since IDMseq sequences individual original molecules, it necessitates that the Illumina and Nanopore experiments sequenced two distinct subsets of the original pool of molecules. It is possible that these three SNVs had lower actual allele frequencies (below the nominal 1/15598 or 6.4x10-5) and happened to be present in the subset of original molecules that went into the Nanopore library but not the Illumina one. As with any high-throughput sequencing method, the accuracy of allele frequency estimate improves with sequencing depth. Because of the high cost of Nanopore sequencing, it was performed at a depth that was enough to analyze the knock-in SNV (approximately 1/8 of the depth of the Illumina sequencing), which was the original goal of this experiment. However, this sequencing depth might not be enough for the analysis of ultra-rare somatic mutations, so these Nanopore somatic mutation data should be interpreted with caution. Nevertheless, the overall calculated somatic SNV load in the Illumina sequencing was 7.1 per Mb, which closely matched the Nanopore data (Table 1). The sensitivity and accuracy of IDMseq in detecting SNVs and estimating variant frequency were demonstrated using the introduced SNV. As shown in Fig 1g, the detected variant frequency closely matched the introduced value in different scenarios (1:100, 1:1000, 1:10,000, on Illumina, Nanopore, and PacBio platforms). Based on these findings, we believe the 3 non-overlapping SNVs are less likely due to ~30% error rates of VAULT, but rather because of stochastic partition of molecules in the sequencing libraries and overestimation of VAF due to insufficient sequencing depth. We have added the above discussion in the revised manuscript on Page 4, line 33.*

3) Page 5, line 3. "Functional annotation of the SNVs showed that 17 of 192 caused an amino acid change." The number (192) doesn’t fit what is shown in the table of Fig. 1d.

*Reply: We thank the reviewer for pointing this out. We want to clarify that the number in the “Count” column represent annotation record count, which is different from unique SNV count. The SNV annotation in Fig. 1d was done using SnpEff v4.3 with the hg38 genome annotation. It annotates SNVs based on the transcripts they are in, and for each transcript, it will generate one annotation record. The EPOR gene has 4 transcripts in hg38, and thus generates more annotation records than the total SNV number (192). We have added the method of SNV annotation on Page 11, line 16, and an explanation of this table in the figure legend on Page 16, line 18.*

**Third round of review**

**Reviewer 2**

The authors have addressed all my concerns.
